# Supplementary material for: Phenylpropanoid Content of Chickpea Seed Coats in Relation to Seed Dormancy
Source: Plants (Basel). 2023 Jul 19;12(14):2687. doi: 10.3390/plants12142687 (PMC10384132; doi:10.3390/plants12142687)
Supplement: Supplementary file 1 [file plants-12-02687-s001.zip › Supplementary table 1A,B.pdf]

Supplementary table 1A – Average values of phenolic compounds (pmol/mg DW) detected in the seed coats of non-dormant RILs.

Class	Compound	CRIL2-5		CRIL2-6		CRIL2-7		CRIL2-15	
		Mean	SD	Mean	SD	Mean	SD	Mean	SD
Hydroxybenzoate	Gallic acid	14.240	0.968	10.705	1.199	1.278	0.406	2.353	0.411
	Salicylic acid-2-O-β-D-glucoside	0.110	0.015	0.208	0.026	2.728	0.382	3.005	0.266
	4-Hydroxybenzoic acid	3.263	0.321	2.163	0.277	11.239	1.762	6.996	0.656
	Vanillic acid	–	–	–	–	0.661	0.101	–	–
	3-Hydroxybenzoic acid	0.064	0.012	0.032	0.005	0.136	0.013	0.059	0.006
	Syringic acid	0.049	0.008	0.055	0.003	0.022	0.005	0.020	0.004
	Salicylic acid	0.244	0.051	0.286	0.031	1.670	0.220	0.875	0.122
Hydroxycinnamate	Chlorogenic acid	0.204	0.035	0.198	0.048	0.144	0.019	0.351	0.036
	Caffeic acid	0.022	0.001	0.036	0.003	0.033	0.002	0.055	0.011
	p-Coumaric acid	0.214	0.004	0.427	0.060	0.274	0.026	0.296	0.025
	Ferulic acid	0.657	0.060	0.965	0.176	0.443	0.017	0.460	0.045
	Sinapic acid	0.113	0.014	0.309	0.046	0.206	0.012	0.097	0.004
Hydroxycinnamic acid derivatives	Coniferaldehyde	0.063	0.005	0.069	0.005	0.028	0.003	0.050	0.008

		CRIL2-23		CRIL2-43		CRIL2-45		CRIL2-47	
		Mean	SD	Mean	SD	Mean	SD	Mean	SD
Hydroxybenzoate	Gallic acid	2.223	0.193	5.415	0.445	0.673	0.032	2.054	0.099
	Salicylic acid-2-O-β-D-glucoside	4.522	0.226	2.882	0.557	6.013	0.670	1.797	0.199
	4-Hydroxybenzoic acid	7.497	0.833	10.130	0.707	8.797	1.295	7.617	1.384
	Vanillic acid	0.534	0.123	1.225	0.107	0.552	0.048	0.487	0.133
	3-Hydroxybenzoic acid	0.145	0.016	0.119	0.019	0.073	0.023	0.082	0.021
	Syringic acid	0.050	0.013	0.051	0.002	0.022	0.003	0.041	0.005
	Salicylic acid	2.077	0.173	2.799	0.280	1.786	0.344	0.651	0.229
Hydroxycinnamate	Chlorogenic acid	0.170	0.017	0.433	0.096	0.276	0.059	0.144	0.029
	Caffeic acid	0.018	0.003	0.038	0.005	0.038	0.006	0.032	0.006
	p-Coumaric acid	0.157	0.023	0.209	0.025	0.210	0.035	0.234	0.053
	Ferulic acid	0.217	0.014	1.210	0.152	0.480	0.092	0.538	0.089
	Sinapic acid	0.195	0.013	0.478	0.053	0.054	0.012	0.571	0.133
Hydroxycinnamic acid derivatives	Coniferaldehyde	0.029	0.005	0.080	0.004	0.033	0.004	0.016	0.002

Supplementary table 1B – Average values of phenolic compounds (pmol/mg DW) detected in the seed coats of non-dormant RILs.

Class	Compound	CRIL2-50		CRIL2-51		CRIL2-65		CRIL2-80	
		Mean	SD	Mean	SD	Mean	SD	Mean	SD
Hydroxybenzoate	Gallic acid	1.311	0.375	3.482	0.384	5.834	0.296	1.836	0.273
	Salicylic acid-2-O-β-D-glucoside	8.148	1.557	1.277	0.169	1.106	0.260	2.858	0.351
	4-Hydroxybenzoic acid	6.311	0.796	3.714	0.407	8.678	1.286	10.778	1.939
	Vanillic acid	0.476	0.103	–	–	0.878	0.248	–	–
	3-Hydroxybenzoic acid	0.122	0.011	0.101	0.039	0.124	0.009	0.224	0.030
	Syringic acid	0.019	0.003	0.034	0.004	0.053	0.013	0.066	0.010
	Salicylic acid	2.566	0.391	1.128	0.093	0.766	0.018	2.960	0.824
Hydroxycinnamate	Chlorogenic acid	0.128	0.020	0.191	0.030	0.234	0.060	0.633	0.104
	Caffeic acid	0.036	0.006	0.014	0.001	0.066	0.006	0.031	0.004
	p-Coumaric acid	0.157	0.048	0.172	0.004	0.250	0.067	0.248	0.044
	Ferulic acid	0.452	0.035	0.396	0.023	0.865	0.253	0.588	0.118
	Sinapic acid	0.272	0.086	0.240	0.018	0.194	0.050	0.146	0.029
Hydroxycinnamic acid derivatives	Coniferaldehyde	0.019	0.005	0.060	0.005	0.045	0.012	0.024	0.004
		CRIL2-81		CRIL2-89		CRIL2-110		CRIL2-111	
		Mean	SD	Mean	SD	Mean	SD	Mean	SD
Hydroxybenzoate	Gallic acid	1.327	0.341	1.190	0.085	1.578	0.502	5.517	0.378
	Salicylic acid-2-O-β-D-glucoside	2.906	0.298	3.643	0.091	1.395	0.257	0.917	0.138
	4-Hydroxybenzoic acid	8.452	0.630	7.759	1.089	8.076	1.797	2.934	0.697
	Vanillic acid	0.828	0.123	0.642	0.135	0.644	0.047	–	–
	3-Hydroxybenzoic acid	0.092	0.038	0.082	0.028	0.097	0.010	–	–
	Syringic acid	0.058	0.015	0.019	0.002	0.060	0.009	0.072	0.010
	Salicylic acid	0.642	0.110	1.110	0.193	0.624	0.081	0.336	0.067
Hydroxycinnamate	Chlorogenic acid	0.505	0.120	0.395	0.081	0.377	0.083	0.360	0.041
	Caffeic acid	0.055	0.017	0.038	0.010	0.031	0.003	0.027	0.002
	p-Coumaric acid	0.204	0.013	0.182	0.021	0.195	0.015	0.106	0.009
	Ferulic acid	1.499	0.164	0.485	0.036	0.933	0.103	0.349	0.014
	Sinapic acid	0.513	0.047	0.398	0.029	0.567	0.201	1.551	0.256
Hydroxycinnamic acid derivatives	Coniferaldehyde	0.015	0.004	0.015	0.003	0.032	0.001	0.028	0.001
