# Supplementary material for: Phenylpropanoid Content of Chickpea Seed Coats in Relation to Seed Dormancy
Source: Plants (Basel). 2023 Jul 19;12(14):2687. doi: 10.3390/plants12142687 (PMC10384132; doi:10.3390/plants12142687)
Supplement: Supplementary file 1 [file plants-12-02687-s001.zip › Supplementary table 2A,B.pdf]

Supplementary table 2A – Average values of phenolic compounds (pmol/mg DW) detected in the seed coats of dormant RILs and intermediate CRIL2-25.

Class	Compound	CRIL2-14		CRIL2-21		CRIL2-27		CRIL2-42	
		**Mean**	**SD**	**Mean**	**SD**	**Mean**	**SD**	**Mean**	**SD**
Hydroxybenzoate	Gallic acid	–	–	–	–	3.837	0.457	–	–
	Salicylic acid-2-O-β-D-glucoside	4.275	0.326	1.606	0.186	2.448	0.071	2.750	0.110
	4-Hydroxybenzoic acid	4.518	0.407	6.772	0.478	4.828	0.304	5.622	0.231
	Vanillic acid	0.389	0.011	1.904	0.098	–	–	0.907	0.056
	3-Hydroxybenzoic acid	0.134	0.032	0.185	0.007	0.077	0.006	0.148	0.020
	Syringic acid	0.028	0.003	0.027	0.004	0.048	0.004	0.024	0.001
	Salicylic acid	1.304	0.116	0.396	0.064	0.987	0.145	0.765	0.101
Hydroxycinnamate	Chlorogenic acid	0.374	0.012	0.238	0.018	0.103	0.015	0.324	0.049
	Caffeic acid	0.016	0.004	0.012	0.002	0.019	0.003	0.037	0.005
	p-Coumaric acid	0.140	0.011	0.157	0.023	0.210	0.015	0.204	0.005
	Ferulic acid	0.415	0.034	1.949	0.529	0.329	0.088	1.991	0.164
	Sinapic acid	0.140	0.010	0.370	0.049	0.176	0.004	0.269	0.020
Hydroxycinnamic acid derivatives	Coniferaldehyde	0.044	0.003	0.014	0.002	0.049	0.016	0.074	0.017

		CRIL2-48		CRIL2-60		CRIL2-79		CRIL2-106	
		**Mean**	**SD**	**Mean**	**SD**	**Mean**	**SD**	**Mean**	**SD**
Hydroxybenzoate	Gallic acid	1.336	0.231	–	–	–	–	0.297	0.015
	Salicylic acid-2-O-β-D-glucoside	3.641	0.456	4.936	0.391	2.639	0.181	5.787	0.489
	4-Hydroxybenzoic acid	4.984	0.555	12.974	0.798	7.461	0.527	12.640	1.319
	Vanillic acid	0.500	0.039	1.236	0.125	0.636	0.036	1.302	0.141
	3-Hydroxybenzoic acid	0.069	0.012	0.115	0.023	0.100	0.008	0.153	0.047
	Syringic acid	0.016	0.002	0.018	0.000	0.023	0.002	0.021	0.001
	Salicylic acid	1.489	0.143	0.927	0.105	0.989	0.237	1.284	0.242
Hydroxycinnamate	Chlorogenic acid	0.307	0.065	0.648	0.140	0.357	0.060	0.806	0.168
	Caffeic acid	0.050	0.015	0.095	0.011	0.022	0.001	0.088	0.008
	p-Coumaric acid	0.290	0.093	0.446	0.029	0.253	0.028	0.372	0.054
	Ferulic acid	0.665	0.138	1.208	0.087	0.457	0.054	0.981	0.128
	Sinapic acid	0.064	0.011	0.120	0.014	0.215	0.019	0.097	0.009
Hydroxycinnamic acid derivatives	Coniferaldehyde	0.095	0.014	0.073	0.003	0.020	0.004	0.051	0.010

Supplementary table 2B – Average values of phenolic compounds (pmol/mg DW) detected in the seed coats of dormant RILs and intermediate CRIL2-25.

Class	Compound	CRIL2-114		CRIL2-115		CRIL2-129		CRIL2-131	
		**Mean**	**SD**	**Mean**	**SD**	**Mean**	**SD**	**Mean**	**SD**
Hydroxybenzoate	Gallic acid	2.100	0.314	–	–	–	–	–	–
	Salicylic acid-2-O-β-D-glucoside	5.056	0.459	1.961	0.292	3.845	0.350	4.802	0.080
	4-Hydroxybenzoic acid	8.243	0.984	6.008	1.401	12.657	1.219	12.760	0.105
	Vanillic acid	–	–	0.407	0.139	0.737	0.087	1.459	0.082
	3-Hydroxybenzoic acid	0.179	0.040	0.208	0.040	0.173	0.031	0.214	0.032
	Syringic acid	0.035	0.009	0.015	0.001	0.022	0.001	0.030	0.007
	Salicylic acid	2.291	0.231	0.673	0.104	1.087	0.123	1.549	0.038
Hydroxycinnamate	Chlorogenic acid	0.588	0.061	0.384	0.057	0.500	0.112	0.390	0.138
	Caffeic acid	0.029	0.008	0.028	0.001	0.079	0.018	0.035	0.005
	p-Coumaric acid	0.248	0.053	0.207	0.030	0.380	0.050	0.294	0.006
	Ferulic acid	0.303	0.037	0.834	0.144	1.335	0.149	1.016	0.011
	Sinapic acid	0.053	0.012	0.092	0.029	0.193	0.022	0.151	0.001
Hydroxycinnamic acid derivatives	Coniferaldehyde	0.064	0.007	0.015	0.005	0.063	0.007	0.059	0.001

		CRIL2-25	
		**Mean**	**SD**
Hydroxybenzoate	Gallic acid	7.581	0.562
	Salicylic acid-2-O-β-D-glucoside	4.547	0.600
	4-Hydroxybenzoic acid	5.288	0.502
	Vanillic acid	–	–
	3-Hydroxybenzoic acid	0.101	0.014
	Syringic acid	0.051	0.006
	Salicylic acid	2.147	0.345
Hydroxycinnamate	Chlorogenic acid	0.490	0.111
	Caffeic acid	0.013	0.001
	p-Coumaric acid	0.176	0.022
	Ferulic acid	0.358	0.040
	Sinapic acid	0.117	0.012
Hydroxycinnamic acid derivatives	Coniferaldehyde	0.072	0.008
