# Supplementary material for: Phenylpropanoid Content of Chickpea Seed Coats in Relation to Seed Dormancy
Source: Plants (Basel). 2023 Jul 19;12(14):2687. doi: 10.3390/plants12142687 (PMC10384132; doi:10.3390/plants12142687)
Supplement: Supplementary file 1 [file plants-12-02687-s001.zip › Supplementary table 3A,B.pdf]

Supplementary table 3A – Average values of flavonoids (pmol/mg DW) detected in the seed coats of non-dormant RILs.

Class	Compound	CRIL2-5		CRIL2-6		CRIL2-7		CRIL2-15	
		**Mean**	**SD**	**Mean**	**SD**	**Mean**	**SD**	**Mean**	**SD**
Flavanol	Gallocatechin	9.284	0.154	17.630	1.918	11.878	1.964	19.461	2.354
	Catechin	–	–	–	–	0.587	0.067	–	–
Flavonol	Myricetin	8.468	1.037	12.550	0.594	6.546	1.709	20.010	1.134
	Quercetin	0.163	0.014	0.089	0.014	0.637	0.049	0.193	0.017
	Kaempferol	0.050	0.014	0.027	0.008	0.466	0.044	0.036	0.006
	Morin	3.329	0.647	2.664	0.297	8.807	2.385	4.837	0.436
	Myricitrin	1.266	0.213	1.338	0.170	0.894	0.091	2.202	0.203
	Quercitrin	1.532	0.085	0.658	0.063	2.447	0.327	1.951	0.287
Flavone	Luteolin	0.810	0.181	0.446	0.027	1.925	0.117	0.912	0.115
	Isoorientin	0.015	0.003	0.023	0.003	0.034	0.006	0.009	0.001
	Orientin	0.029	0.003	0.047	0.009	0.060	0.017	0.020	0.002
	Isovitexin	0.041	0.011	0.005	0.001	0.001	0.000	0.008	0.001
Flavonone	Naringenin	4.220	0.335	8.754	1.078	5.347	0.950	4.252	0.947
		**CRIL2-23**		**CRIL2-43**		**CRIL2-45**		**CRIL2-47**	
		**Mean**	**SD**	**Mean**	**SD**	**Mean**	**SD**	**Mean**	**SD**
Flavanol	Gallocatechin	10.860	0.991	12.223	0.861	12.528	2.808	6.619	1.098
	Catechin	0.826	0.081	0.688	0.072	–	–	0.917	0.121
Flavonol	Myricetin	6.626	0.781	9.674	2.276	9.437	1.932	4.879	0.630
	Quercetin	0.773	0.068	0.977	0.255	0.047	0.008	0.598	0.039
	Kaempferol	0.467	0.056	0.584	0.080	0.024	0.003	0.635	0.141
	Morin	13.068	0.716	15.149	2.875	0.584	0.173	12.072	1.157
	Myricitrin	0.832	0.030	1.469	0.195	2.541	0.390	0.621	0.106
	Quercitrin	3.800	0.087	4.499	0.769	2.506	0.383	2.597	0.436
Flavone	Luteolin	3.275	0.274	3.128	0.645	0.603	0.071	1.371	0.164
	Isoorientin	0.020	0.001	0.007	0.002	0.010	0.001	0.009	0.003
	Orientin	0.043	0.010	0.022	0.003	0.031	0.007	0.031	0.007
	Isovitexin	0.003	0.002	0.001	0.000	0.005	0.001	0.002	0.001
Flavonone	Naringenin	8.826	0.571	7.797	1.869	3.017	0.683	4.873	0.797

Supplementary table 3B – Average values of flavonoids (pmol/mg DW) detected in the seed coats of non-dormant RILs..

Class	Compound	CRIL2-50		CRIL2-51		CRIL2-65		CRIL2-80	
		**Mean**	**SD**	**Mean**	**SD**	**Mean**	**SD**	**Mean**	**SD**
Flavanol	Gallocatechin	7.278	1.913	11.693	0.480	6.366	0.241	7.775	1.917
	Catechin	0.421	0.147	0.606	0.157	–	–	–	–
Flavonol	Myricetin	2.542	0.481	11.964	1.703	8.135	1.746	10.071	1.704
	Quercetin	0.174	0.044	0.570	0.079	0.029	0.002	0.042	0.010
	Kaempferol	0.033	0.008	0.298	0.030	0.014	0.002	0.020	0.002
	Morin	4.648	0.999	11.589	1.102	0.213	0.040	0.109	0.008
	Myricitrin	0.827	0.263	1.379	0.163	1.669	0.404	1.715	0.200
	Quercitrin	3.174	0.670	4.510	0.439	0.632	0.129	1.477	0.358
Flavone	Luteolin	1.727	0.159	2.031	0.244	0.176	0.009	0.209	0.029
	Isoorientin	0.015	0.003	0.006	0.001	–	–	0.010	0.002
	Orientin	0.028	0.006	–	–	0.026	0.005	0.040	0.007
	Isovitexin	0.002	0.001	0.005	0.000	0.004	0.001	0.003	0.000
Flavonone	Naringenin	2.676	0.633	5.295	0.970	3.194	0.709	2.678	0.354
		**CRIL2-81**		**CRIL2-89**		**CRIL2-110**		**CRIL2-111**	
		**Mean**	**SD**	**Mean**	**SD**	**Mean**	**SD**	**Mean**	**SD**
Flavanol	Gallocatechin	7.238	0.735	5.916	0.417	5.720	0.576	8.221	0.220
	Catechin	–	–	0.844	0.048	0.894	0.089	0.866	0.142
Flavonol	Myricetin	5.956	0.936	6.094	0.503	3.890	0.390	6.269	1.007
	Quercetin	0.016	0.002	0.259	0.024	0.143	0.031	0.419	0.053
	Kaempferol	0.006	0.001	0.475	0.053	0.321	0.048	0.514	0.156
	Morin	0.957	0.110	7.310	0.473	4.318	0.555	9.629	1.595
	Myricitrin	1.335	0.376	1.072	0.179	0.904	0.254	0.581	0.021
	Quercitrin	1.302	0.280	3.033	0.195	2.175	0.606	2.359	0.547
Flavone	Luteolin	0.126	0.034	0.781	0.089	0.477	0.109	1.519	0.063
	Isoorientin	0.003	0.000	0.008	0.002	0.004	0.001	0.056	0.011
	Orientin	0.018	0.003	–	–	0.017	0.002	0.096	0.015
	Isovitexin	0.002	0.000	0.012	0.002	0.002	0.001	0.011	0.001
Flavonone	Naringenin	2.042	0.185	6.564	0.551	3.150	0.516	7.532	1.311
