# Supplementary material for: Phenylpropanoid Content of Chickpea Seed Coats in Relation to Seed Dormancy
Source: Plants (Basel). 2023 Jul 19;12(14):2687. doi: 10.3390/plants12142687 (PMC10384132; doi:10.3390/plants12142687)
Supplement: Supplementary file 1 [file plants-12-02687-s001.zip › Supplementary table 4A,B.pdf]

Supplementary table 4A – Average values of flavonoids (pmol/mg DW) detected in the seed coats of dormant RILs and intermediate CRIL2-25.

**Class**	**Compound**	**CRIL2-14**		**CRIL2-21**		**CRIL2-27**		**CRIL2-42**	
		**Mean**	**SD**	**Mean**	**SD**	**Mean**	**SD**	**Mean**	**SD**
Flavanol	Gallocatechin	11.506	0.313	5.621	1.072	7.194	1.025	14.633	0.894
	Catechin	–	–	1.587	0.397	–	–	–	–
Flavonol	Myricetin	7.033	0.055	2.251	0.614	7.911	2.017	7.160	1.857
	Quercetin	0.074	0.006	0.331	0.016	0.048	0.014	0.038	0.009
	Kaempferol	0.027	0.003	0.531	0.075	0.035	0.001	0.016	0.003
	Morin	2.497	0.058	6.534	1.876	2.063	0.518	1.668	0.282
	Myricitrin	0.521	0.040	0.393	0.118	0.716	0.216	1.633	0.126
	Quercitrin	0.877	0.143	1.868	0.494	0.635	0.207	2.035	0.078
Flavone	Luteolin	0.752	0.018	1.541	0.221	0.515	0.020	0.436	0.085
	Isoorientin	0.073	0.009	0.022	0.005	0.018	0.001	0.008	0.000
	Orientin	0.151	0.020	0.061	0.013	0.045	0.001	0.034	0.004
	Isovitexin	0.008	0.002	0.002	0.001	0.003	0.001	0.002	0.000
Flavonone	Naringenin	3.368	0.191	3.141	0.644	3.619	0.386	1.814	0.279
		**CRIL2-48**		**CRIL2-60**		**CRIL2-79**		**CRIL2-106**	
		**Mean**	**SD**	**Mean**	**SD**	**Mean**	**SD**	**Mean**	**SD**
Flavanol	Gallocatechin	16.731	2.832	14.893	1.868	12.680	0.934	12.083	1.107
	Catechin	1.137	0.292	0.452	0.038	–	–	–	–
Flavonol	Myricetin	7.459	2.711	11.566	1.566	8.212	0.423	9.894	0.743
	Quercetin	0.751	0.173	0.048	0.009	0.020	0.003	0.035	0.005
	Kaempferol	0.320	0.041	0.016	0.002	0.007	0.001	0.019	0.002
	Morin	11.932	2.706	2.128	0.268	0.163	0.041	1.734	0.180
	Myricitrin	1.305	0.285	1.536	0.065	1.726	0.184	1.153	0.105
	Quercitrin	3.163	1.224	1.527	0.137	1.190	0.059	1.454	0.170
Flavone	Luteolin	5.925	1.507	0.337	0.091	0.343	0.079	1.511	0.118
	Isoorientin	0.053	0.010	0.007	0.001	0.003	0.001	0.021	0.001
	Orientin	0.053	0.015	0.048	0.004	0.023	0.003	0.054	0.006
	Isovitexin	0.005	0.001	0.003	0.000	0.002	0.001	0.006	0.000
Flavonone	Naringenin	4.880	0.867	2.115	0.366	1.958	0.320	2.739	0.234

Supplementary table 4B – Average values of flavonoids (pmol/mg DW) detected in the seed coats of dormant RILs and intermediate CRIL2-25.

Class	Compound	CRIL2-114		CRIL2-115		CRIL2-129		CRIL2-131	
		Mean	SD	Mean	SD	Mean	SD	Mean	SD
Flavanol	Gallocatechin	8.995	0.844	7.442	1.885	12.640	0.831	17.576	0.225
	Catechin	–	–	–	–	0.558	0.125	0.518	0.051
Flavonol	Myricetin	10.519	1.303	4.367	0.169	15.143	2.349	9.365	0.305
	Quercetin	0.040	0.014	0.035	0.005	0.082	0.014	0.040	0.003
	Kaempferol	0.026	0.005	0.014	0.002	0.024	0.003	0.019	0.002
	Morin	0.134	0.028	0.114	0.026	3.329	0.218	0.210	0.012
	Myricitrin	0.424	0.050	0.531	0.152	3.049	0.129	1.472	0.021
	Quercitrin	0.705	0.094	0.651	0.228	2.958	0.196	0.900	0.082
Flavone	Luteolin	0.441	0.106	0.627	0.003	0.495	0.031	0.571	0.051
	Isoorientin	0.036	0.010	0.010	0.000	0.009	0.002	0.009	0.001
	Orientin	–	–	0.030	0.004	0.026	0.008	0.017	0.001
	Isovitexin	0.005	0.001	0.002	0.000	0.004	0.001	0.004	0.001
Flavonone	Naringenin	2.652	0.510	2.936	0.703	3.699	0.628	3.534	0.297

		CRIL2-25	
		Mean	SD
Flavanol	Gallocatechin	9.642	0.929
	Catechin	–	–
Flavonol	Myricetin	10.735	0.919
	Quercetin	0.094	0.013
	Kaempferol	0.043	0.001
	Morin	2.954	0.445
	Myricitrin	0.834	0.093
	Quercitrin	0.825	0.055
Flavone	Luteolin	0.831	0.109
	Isoorientin	0.052	0.006
	Orientin	0.114	0.016
	Isovitexin	0.006	0.002
Flavonone	Naringenin	3.467	0.765
